# Association between universal health coverage and the disease burden of acute illness and injury at the global level

**DOI:** 10.1186/s12889-023-15671-2

**Published:** 2023-04-21

**Authors:** Karim Hajjar, Luis Lillo, Diego A Martinez, Manuel Hermosilla, Nicholas Risko

**Affiliations:** 1 Department of Emergency Medicine, Johns Hopkins University School of Medicine, Baltimore, USA; 2 School of Industrial Engineering, Pontificia Universidad Católica de Valparaíso, Valparaíso, Chile; 3 Johns Hopkins Carey Business School, Baltimore, USA

**Keywords:** Emergency care, Emergency care systems, Global burden of disease, Catastrophic health expenditure, Sustainable development goals

## Abstract

**Background:**

This study examines the relationship between universal health coverage (UHC) and the burden of emergency diseases at a global level.

**Methods:**

Data on Disability-Adjusted Life Years (DALYs) from emergency conditions were extracted from the Institute for Health Metrics and Evaluation (IHME) database for the years 2015 and 2019. Data on UHC, measured using two variables 1) coverage of essential health services and 2) proportion of the population spending more than 10% of household income on out-of-pocket health care expenditure, were extracted from the World Bank Database for years preceding our outcome of interest. A linear regression was used to analyze the association between UHC variables and DALYs for emergency diseases, controlling for other variables.

**Results:**

A total of 132 countries were included. The median national coverage of essential health services index was 67.5/100, while the median national prevalence of catastrophic spending in the sample was 6.74% of households. There was a strong significant relationship between health service coverage and the burden of emergency diseases, with an 11.5-point reduction in DALYs of emergency medical diseases (95% CI -9.5, -14.8) for every point increase in the coverage of essential health services index. There was no statistically significant relationship between catastrophic expenditures and the burden of emergency diseases, which may be indicative of inelastic demand in seeking services for health emergencies.

**Conclusion:**

Increasing the coverage of essential health services, as measured by the essential health services index, is strongly correlated with a reduction in the burden of emergency conditions. In addition, data affirms that financial protection remains inadequate in many parts of the globe, with large numbers of households experiencing significant economic duress related to seeking healthcare. This evidence supports a strategy of strengthening UHC as a means of combating death and disability from health emergencies, as well as extending protection against impoverishment related to healthcare expenses.

**Supplementary Information:**

The online version contains supplementary material available at 10.1186/s12889-023-15671-2.

## Background

All United Nations (UN) member states have agreed to work towards achieving 17 sustainable development goals (SDGs) with 169 targets, by the year 2030. These goals aim to achieve a world free from poverty, hunger, and disease. SDG target 3 centers around health with the aim to “Ensure healthy lives and promote well-being for all at all ages” and is underpinned by 13 targets. Target 3.8 aims to “Achieve universal health coverage, including financial risk protection, access to quality essential healthcare services and access to safe, effective, quality and affordable essential medicines and vaccines for all” [1]. According to the World Health Organization (WHO), Universal Health Coverage (UHC) means that, “all people have access to the health services they need, when and where they need them, without financial hardship” [2]. Governments and policies play a central role in achieving UHC [3].

While 75% of national health policies and strategies are aimed at moving towards UHC, accelerated progress towards the realization of UHC is needed. According to the WHO, at least half of the people in the world cannot receive the services they need, over 930 million people spend over 10% of their income on health care, and 100 million are driven into poverty each year through out-of-pocket health spending [2]. Measuring progress on UHC can also be challenging. To this end, the WHO and the World Bank have developed a framework to track a nation’s progress towards UHC, focusing on two indicators: access to essential quality health services and protection from financial hardship [4].

Acute unplanned illnesses and injuries happen everywhere daily, whether there is an organized and accessible health system to address them or not. These emergency health conditions significantly contribute to out-of-pocket spending, bankruptcy and poverty across the world [5]. Emergency conditions also make up a significant part of the global burden of disease [6, 7]. Improving access to quality emergency treatment has the potential to prevent death and disability around the world, and this has been enshrined as a global health priority in multiple World Health Assembly declarations [8].

While emergency care systems and services are a critical component of UHC, the relationship between progress on UHC, as measured by the WHO’s statistical approach to quantifying UHC, and emergency disease outcomes remains unstudied [9]. Our research aims to analyze the relationship between universal healthcare and the burden of emergency diseases on a global scale.

## Methods

### Study design

Our study was a retrospective population-based analytical study, examining the relationship between universal health coverage through two independent variables and the burden of emergency diseases on a global level.

### Inclusion/exclusion criteria

Countries with missing data were excluded. Population and health data were obtained for the 193 UN member states from various sources, with the information distributed between 2005 and 2019. Sixty-one were excluded from the analysis due to missing variables. A total of 132 countries were included in our analysis.

### Data source (Additional file 1: Appendix 1)

#### Universal healthcare coverage: independent variables

We used the framework developed by the WHO and the World Bank to determine each country’s UHC level, a composite of two indicators: the coverage of essential health services or UHC Service Coverage Index (SCI) and catastrophic healthcare expenditure (CHE). UHC SCI is defined as the average coverage of essential services based on tracer interventions that include reproductive, maternal, newborn and child health, communicable and non-communicable diseases, and healthcare service capacity and access among the general and the most disadvantaged population [10]. The indicator is an index reported on a unitless scale of 0 to 100, computed as the geometric mean of 14 tracer indicators of UHC SCI. The WHO reported UHC SCI for 2015 and 2017; we used the arithmetic mean between 2015 and 2017 for each included country. CHE is the proportion of the population with large household expenditure on health as a share of total household income [11]. Two thresholds are reported as large household expenditures; greater than 10% and greater than 25%. In our study, we used the 10% threshold to identify more households with increased health expenditures. UHC information was extracted from the WHO and World Bank databases for 132 countries (61 countries with no reported data) between 2005 and 2015.

#### Burden of emergency diseases: dependent variable

We used disability-adjusted life year (DALY) for emergency medical conditions as a surrogate for the burden of emergency diseases. The DALY is a summary public health measure commonly used to quantify disease burden. This burden represents the gap between a population’s ideal health and its current health status. It measures the losses in healthy life years caused by living with illness and/or through dying before an expected life expectancy [12].

The DALYs were collected from the Institute for Health Metrics and Evaluation (IHME) database. We calculated the arithmetic mean DALY of every emergency medical condition per country, over the 5 years that came after the year of exposure. These emergency medical conditions that were selected were diseases that, if not diagnosed and treated within hours or days, commonly lead to serious physical or mental disability or death or conditions with trajectories that can involve acute decompensation that lead many patients to experience severe physical or mental disability or death [7]. We included 43 conditions that met these definitions (Table 1).

**Table 1:** Emergency medical conditions

Acute glomerulonephritis	Glaucoma	Pulmonary aspiration and foreign body in airway
Acute hepatitis	Idiopathic epilepsy	Rabies
Animal contact	Interpersonal violence	Road injuries
Appendicitis	Ischemic heart disease	Self-harm
Atrial fibrillation and flutter	Lower respiratory infections	Self-harm and interpersonal violence
Cardiomyopathy and myocarditis	Malaria	Stroke
Cellulitis	Maternal disorders	Sudden infant death syndrome
Dengue	Measles	Tetanus
Diarrheal diseases	Meningitis	Transport injuries
Diphtheria	Neonatal disorders	Typhoid and paratyphoid
Encephalitis	Other transport injuries	Unintentional injuries
Endocarditis	Other unintentional injuries	Varicella and herpes zoster
Environmental heat and cold exposure	Paralytic ileus and intestinal obstruction	Whooping cough
Exposure to forces of nature	Poisonings	Yellow fever
Exposure to mechanical forces		

#### Covariates

We included demographic, socioeconomic, and health-related variables as covariates. The variables included were gross domestic product (GDP) per capita, population aged 65 and older, and physicians and hospital beds per 1,000 inhabitants. The information was obtained from various sources (Supplementary table) between 2005 and 2019.

#### Data analysis

The independent variables were UHC SCI and CHE for each country. The dependent variable was DALY for the 43 emergency medical conditions. Covariates included the population and health data variables. The Spearman correlation test was used to assess if any of the independent variables and covariates were colinear, with a maximum correlation level tolerance of 0.8.

The association between UHC and the burden of emergency diseases was analyzed using unadjusted and adjusted linear regressions. For the dependent variable, the average DALYs for the 5 years following the year of exposure to UHC SCI and CHE was used. For UHC SCI, the year of exposure utilized was 2015. For CHE, the year of exposure utilized was the year with the most recent data available between 2005 and 2015. Models were adjusted for GDP per capita, population aged 65 and older, and physicians and hospital beds per 1,000 inhabitants. For covariates, the value corresponding to the year of exposure of each independent variable was used. We determined the values of R-squared, and regression coefficients with a confidence interval of 95%. The P-value was determined, and the result of this test was considered statistically significant if it was less than 0.05.

## Results

In our study, 132 countries were included to examine the association between universal health coverage and emergency care outcomes. Forty-three countries were African (32.6%), 35 were European (26.5%), 31 were Asian (23.5%), 17 were South-American (12.9%), 3 were North-American (2.3%), and 2 were Oceanic (1.5%).

Table 2 shows the baseline characteristics of the countries under study. The median DALYs from emergency conditions was 398.45 per 100,000 population and was highest in low-income countries (901.71) and lowest in high-income countries (219.82). The countries with the highest average DALYs were the Central African Republic (1585.8 per 100,000), Niger (1456.0 per 100,000) and Guinea (1153.4 per 100,000). Those with the lowest DALYs were Israel (135.8 per 100,000), Singapore (138.8 per 100,000) and Ireland (165.4 per 100,000) (Appendix 2).

**Table 2:** Summary statistics of the catastrophic health expenditure, coverage of essential health services, burden of disease, and covariates across all countries

	**All**	**High-income**	**Upper middle-income**	**Lower middle-income**	**Low-income**
	*Median (Q1, Q3)*				
Catastrophic Health Expenditure %^b^	6.75 [3.30—12.82]	6.04 [3.27—11.78]	8.07 [3.12—13.64]	7.76 [3.79—13.39]	6.63 [3.22—10.65]
Universal Health Coverage Service Coverage Index %^b^	67.5 [49.5—75.5]	79.00 [74.5—82.5]	69.00 [63.5—75.00]	54.00 [46.50—65.50]	39.00 [35.75—43.75]
GDP per capita (USD)	10,250 [ 3,347—22,590]	36,883 [27,888—46,534]	12,829 [10,130—15,131]	4,250 [2,784—6,421]	1,493 [1,076—1,815]
Physicians per 1,000^a^	1.23 [0.26—2.51]	2.86 [2.22—3.64]	1.76 [0.75—2.45]	0.37 [0.17—0.79]	0.08 [0.04—3.02]
Hospital beds per 1,000^a^	2.32 [1.15—3.86]	3.58 [2.71—5.83]	3.00 [1.94—4.03]	1.45 [0.90—2.26]	0.83 [0.50—1.12]
Population aged 65 and older %^a^	5.54 [3.29—12.27]	15.04[10.51—18.04]	7.23 [5.39—10.52]	4.14 [3.15—5.14]	2.76 [2.58—3.02]
DALYs per 100,000 pop^c^	398.45 [274.97—611.67]	219.82 [191.82—303.34]	349.29 [278.62—496.47]	536.35 [409.54—688.23]	901.71 [714.11—1,230.07]

*Abbreviations*: *GDP* Gross domestic product, *DALYs* Disability adjusted life years

^a^The value for the year of exposure is used

^b^Corresponds to the value of the year with the most recent information up to the year 2015

^c^Average value of the outcome of the 5 years following the year of exposure

There was a similar trend with the UHC SCI variable. Low-income countries had a median UHC SCI of 39 and high-income countries had a median of 79 with all countries having a median of 67.5 (average of years 2015 and 2017) (Table 2). The countries with the highest coverage of essential health services were Canada (89/100), Australia (87/100) and the United Kingdom (86/100). Madagascar (26/100) and South Sudan (31/100) had the lowest coverage (Additional file 2: Appendix 2).

For CHE, the highest medians were observed in the upper and lower middle-income categories (8.07% and 7.76% respectively), with all countries having a median CHE of 6.75% (Table 2). Interestingly, low-income countries had the lowest mean CHE (6.95%), followed by high-income countries (7.82%) and middle-income countries (9.19%) (Additional file 2: Appendix 2). Georgia (29.2%), Egypt (26.2%) and Brazil (25.6%) had the highest proportion of the population who had greater than 10% of household expenditure on health as a share of total household expenditure or income. Gambia (0.2%) and Zambia (0.3%) had the lowest proportion (Additional file 2: Appendix 2).

Of the countries included, 77 (58.3%) had a GDP per capita above $10,000 and 36 (27.3%) were considered high-income countries. The average GDP per capita (years 2015 and 2017) for the countries analyzed was $17,579 (Additional file 2: Appendix 2).

Table 3 represents a linear regression model analyzing the effects of the independent variables a) coverage of essential health services and b) proportion of population with large household expenditure on DALYs of emergency medical diseases, adjusting for the above-mentioned confounding variables. Table 3 shows that for every point increase in the coverage of essential health services index, there was an 11.5 (95% confidence interval [CI] -14.8, -9.5) point reduction in DALYs of emergency medical diseases. Table 3 also demonstrates that for every percent decrease of the population with large household expenditure on healthcare, there was a 1.93 increase in DALYs of emergency medical diseases (95% CI -6.8, 2.9), meaning there was no statistically-significant relationship between catastrophic expenditures and emergency DALYs.

**Table 3:** Adjusted and unadjusted regression models

***Model***		
**Covariates**	**Beta (95% CI)** ^**a**^	**Adj. Beta (95% CI)**^**b**^
*Disability adjusted life years*		
Catastrophic healthcare expenditure	2.59 [-3.9, 9.1]	-1.93 [- 6.8, 2.9]
UHC Service Coverage Index	-12.39 [-13.7, -11.1]*	-11.50 [-14.8, -9.5]*
*UHC Service Coverage Index*		
Healthcare service coverage	-0.007[-0.1, 0.1]	2.04 [0.0, 0.4]*

*Abbreviations: CI *confidence interval, *Adj. *adjusted, *GDP *gross domestic product, *DALYs *Disability adjusted life years, *UHC *Universal Health Coverage

^a^Betas were calculated with a linear regression model

^b^Betas were calculated with linear regression with country-level fixed effects [GDP per capita, physicians per 1,000, hospital beds per 1,000, and percent of population aged 65 and older]

^*^Statistically significant at a *P* < .05 threshold with P values and 95% CI calculated from T-statistic

## Discussion

We performed a global analysis using all available data to examine the relationship between the burden of acute disease and UHC, as measured by two indicators selected by the WHO: coverage of essential health services index and catastrophic health expenditures. To our knowledge, this is the first study of its kind analyzing the association between UHC and the burden of emergency diseases. We focused on acute disease because it represents a substantial but under-recognized global health priority. In fact, emergency medical diseases contribute to 50.7% of mortality and 41.5% of all disease burden globally [6]. This burden is carried disproportionately by lower-income countries and seeking emergency care is often inevitable for patients themselves, regardless of their ability to pay, making it a useful lens through which to examine UHC.

After adjusting for multiple variables, including national income level, increased coverage of essential health services was significantly associated with improvement in DALYs for emergency conditions. There was, however, no association between catastrophic health expenditure and DALYs.

Descriptive analyses were notable for the findings of a correlation between income level, health coverage and disease burden, with lower income countries suffering higher rates of emergency disease and having lower levels of health coverage indices, compared to higher income countries who enjoy high levels of health coverage and low levels of disease burden. Unexpectedly, the lowest-income countries joined the highest income in having the lowest proportion of catastrophic health expenditures, with middle-income countries having the highest.

The positive correlation between income level, health service coverage, and decreasing disease burden is a logical finding that supports a common-sense framework that increased wealth allows for increased investment and access to health services, both preventive and treatment services, which therefore decreases disease burden. The finding of a positive correlation between increased health coverage and lower disease burden even when controlling for income level, supports the assumption that health coverage is the true driver of decreased disease burden, rather than wealth itself.

The lack of a relationship between catastrophic health expenditures and disease burden points to the quality of financial protection being a key intermediary between these variables. Aside from measuring catastrophic health expenditures, the summary statistics measuring UHC do not have another, more direct, assessment of the quality of financial protection. Furthermore, the foreknowledge of being at risk of a heavy financial burden does not strongly mitigate care-seeking behavior in health emergencies. Patients suffering from sudden illness or injury are likely to seek healthcare regardless of ability to pay, for themselves and for their friends and family, reflecting price inelastic demand [13]. This exposes them to catastrophic expenditures in health systems that do not offer financial protection. Further investigation of the underlying factors driving this finding requires future focused research.

When assessing catastrophic expenditures through an income-driven lens, we find that the highest financial burden falls in the middle-income country group. This alludes to a bell-curve relationship where the risk of catastrophic expenditures is highest during the middle-income phase of development where opportunities to purchase services are rapidly expanding but social protections are not. Ostensibly, the lowest income countries have fewer services to physically access, which reduces purchasing/expenditure on those services, while the highest-income countries have enough wealth and financial protection to mitigate catastrophic expenditure. Other researchers have found similar findings, which may also reflect the limitations of heavy reliance on catastrophic health expenditure data to assess financial risk [14]. Regardless, thoroughly investigating the likely causes of this finding was outside the scope of this work and present a prime topic for future research.

There are numerous areas that would benefit from further scholarly work, given their importance to population health and relevance to policy makers. While our study focused on UHC, broader social, political, and economic policies outside the health sector that reduce social inequalities have been shown to have beneficial effects on population health [15–17]. Incorporating standard measures of inequality, such as the Gini Index for example, into analyses on the economic burden of disease and financial protection for health, may provide useful information for high-level decision makers. The lens of the emergency care system could be leveraged for such studies, given its role as a health care safety net for underserved, vulnerable and disenfranchised populations [18, 19].

### Limitations

We encountered several limitations in conducting this research. As is common with health system level global analyses, poor availability of data and vulnerability to confounding were key shortcomings. We utilized careful statistical methods, validated indicators from reliable sources, and research design to reduce bias and provide as much certainty as possible.

The key independent variables driving our statistical model (UHC SCI and CHE) have been extensively validated and operationalized by institutions such as the World Bank and the WHO. They have been selected as the preferred approach to track a nation’s progress towards SDG 3.8 [4]. Despite this, accurately measuring UHC remains a challenge and, as mentioned above, our study ran into previously described limitations with utilizing catastrophic expenditure data as the sole measure of financial risk protection [11]. Other shortcomings of catastrophic expenditure data include data being sourced from a wide range of survey devices, including surveys used to calculate the consumer price index; survey data failing to account for retrospective reimbursement of healthcare spending; and survey data failing to capture hidden debt such as credit card balances, interpersonal loans, or informal payment plans to hospitals [4].

There are shortcomings related to the UHC SCI index as well. Finding regular chronological values for this index for all countries was particularly challenging, for example, data was only available for two years: 2015 and 2017. For this reason, we utilized disease burden data and catastrophic expenditure data corresponding as close as possible to these dates, forcing us to drop some countries that did not have contemporaneous data available or outcome data that immediately followed exposure data. In addition to its sparse temporal coverage, this index is a composite of multiple trace indicators that characterize health service coverage but do not represent an exhaustive list of health services or interventions [10].

Regarding the acute and emergency disease burden, this has been published previously, and we carefully followed previous methods that have been accepted in peer-reviewed literature [6, 7]. Parsing out the proportion of burden measured under a disease heading into its acute versus chronic components is exceedingly difficult and we have relied on the published work of these previous researchers, with its strengths and shortcomings. Lastly, there are many variables that can affect the association between universal health coverage and the burden of emergency diseases. While we incorporated several confounding variables that were also used in other published studies [20], other relevant variables are either missing or difficult to quantify and were thus not included in our analysis.

## Conclusion

This research examined the statistical relationship between measures of UHC and the burden of acute diseases at the global level. We utilized variables identified by the international community as the primary indicators of UHC progress. Our study found that an increase in the coverage of essential health services was significantly associated with a lower burden of emergency disease. This reinforces the expansion of comprehensive health services under UHC as a means of combating death and disability from health emergencies.

We failed to identify an association between catastrophic health expenditure and disease burden. This may partially be explained by the price inelasticity of demand for health services in a severe emergency but may also be related to methodologic issues around using catastrophic health expenditure data as the primary measure of financial risk protection for UHC. Regardless, this research affirms a high economic burden on households in many parts of the globe related to seeking care for emergent health conditions, which further supports a strategy of strengthening UHC to extend financial protection against impoverishment related to health care expenses.

Further research is needed to examine the impact of financial risk protection on acute disease burden and to determine the optimal way of measuring the financial protection components of UHC. Further investigation into how changes in the political and economic determinants of health influence the burden of emergency conditions would also be useful to inform policy and resource allocation.

## Supplementary Information

**Additional file 1: Appendix 1. **Variable definitions and sources.

**Additional file 2: Appendix 2. **Summary of collected variables by country.

## Data Availability

The datasets generated and analyzed during the current study are available in the global burden of disease, [https://www.healthdata.org/gbd/2019] [21], WHO [https://www.who.int/data/collections] [22] and World Bank [https://data.worldbank.org/] [21] repositories.

## Abbreviations

CHE: Catastrophic Health Expenditure
DALY: Disability-Adjusted Life Years
GDP: Gross Domestic Product
IHME: Institute for Health Metrics and Evaluation
SCI: Service Coverage Index
SDG: Sustainable Development Goal
UHC: Universal Health Coverage
UN: United Nations
WHO: World Health Organization
